# DLBCL with amplification of *JAK2/PD-L2* exhibits PMBCL-like CNA pattern and worse clinical outcome resembling those with MYD88 L265P mutation

**DOI:** 10.1186/s12885-020-07293-3

**Published:** 2020-08-27

**Authors:** Xuemin Xue, Wenting Huang, Tian Qiu, Lei Guo, Jianming Ying, Ning Lv

**Affiliations:** 1 Department of Pathology, National Cancer Center/National Clinical Research Center for Cancer/Cancer Hospital, Chinese Academy of Medical Sciences and Peking Union Medical College, Beijing, 100021 China; 2 Department of Pathology, National Cancer Center/National Clinical Research Center for Cancer/Cancer Hospital & Shenzhen Hospital, Chinese Academy of Medical Sciences and Peking Union Medical College, Shenzhen, 518116 China

**Keywords:** Diffuse large B-cell lymphoma, *JAK2*, *PD-L2*, Amplification, Prognosis

## Abstract

**Background:**

Recently, copy number alteration (CNA) of 9p24.1 were demonstrated in 10% of diffuse large b-cell lymphoma (DLBCL), with gene expression and mutation profiles that were similar to those of primary mediastinal large B-cell lymphoma (PMBCL). However, their CNA-based profile and clinical impact still remain unclear.

**Methods:**

Multiplex ligation-dependent probe amplification were employed to investigate the prevalence of *JAK2/PD-L2* amplification in DLBCL and their CNA-based pattern of driver genes. The clinical outcome and characteristics were also analyzed.

**Results:**

Using unsupervised hierarchical clustering, a small group of DLBCL (10.5%, 8/76) was clustered together with PMBCL as Cluster_2, demonstrating amplification of *JAK2* (100%,8/8) and *PD-L2* (75.0%,6/8). This subgroups of DLBCL demonstrated significant higher expression of PD-L1 than those with MYD88 L265P mutation(*p* = 0.024). And they exhibited dismal OS and PFS as compared with DLBCL_others(*p* = 0.003 and 0.001, respectively), which is similar to DLBCL with MYD88 L265P mutation.

**Conclusions:**

DLBCL with amplification of *JAK2/PD-L2* exhibits CNA pattern that is similar to PMBCL, and demonstrates unfavorable clinical outcome that resembles those with MYD88 L265P mutation. It is essential to identify this subgroup of DLBCL who may acquire more benefits from the JAK2 and PD-L1 signaling inhibition.

## Background

Diffuse large B-cell lymphoma (DLBCL) is a highly heterogeneous disease. Recently, several distinctive genetic subtypes were identified, including Schmitz R et al. study (MCD, BN2, N1 and EZB subtypes) and Chapuy B et al. study (C0 ~ C5 clusters) [1, 2]. Godfrey J et al. study also identified an unique biological subset of DLBCL with PD-L1 gene alterations, having high risk features [3]. Thus, the genetics of DLBCL relating to potential therapeutic targets for immune checkpoint inhibitors should be paid much more attention to.

Janus kinase 2 (*JAK2*), programmed cell death 1 ligand 1 (*PD-L1/CD274/PDCD1LG1*) and programmed cell death 1 ligand 2 (*PD-L2*/*CD273/PDCD1LG2*) are adjacent to each other on chromosome 9p24.1, playing key roles in host immune surveillance [4]. Amplification of 9p24.1 were frequently seen in cell lines of classical Hodgkin lymphoma (cHL) (100%, 6/6) and primary mediastinal large B-cell lymphoma (PMBCL) (100%,1/1), but much less in DLBCL cell lines (22%, 4/18, 4]. Correspondingly, PD1 ligands (*PD-L1* and *PD-L2*) transcripts and proteins were more abundant in cHL and PMBCL cell lines than that in DLBCL cell lines [4]. Recently, Y Wang et al. study demonstrated that 10% of DLBCL had copy number alteration (CNA) of 9p24.1, with a gene expression and mutation profile similar to those of PMBCL [5]. However, their CNA-based profile and clinical impact still remain unclear.

Therefore, in this study, we employed multiplex ligation-dependent probe amplification (MLPA) to investigate the prevalence of *JAK2/PD-L2* amplification in DLBCL, and their CNA-based pattern of driver genes, including *BCL2, CDKN2A* and *TP53* [6]. And we analyzed their long-term survival outcome after treatment of R-CHOP-like regime.

## Methods

### Case selection

We collected consecutive cases of DLBCL and PMBCL in our clinical FFPE archives of excisional biopsy database between Jan 2009 and Oct 2010. And 77 cases of DLBCL and 4 cases of PMBCL were found. After confirmation, one case of DLBCL was diagnosed as PMBCL. Thus, 76 cases of DLBCL and 5 cases of PMBCL were acquired finally (see Additional file 1). All patients were diagnosed at National Cancer Center/National Clinical Research Center for Cancer/Cancer Hospital, Chinese Academy of Medical Sciences and Peking Union Medical College according to the revised 4th edition of the WHO Classification of Tumours of Haematopoietic and Lymphoid Tissues [7]. The data regarding treatment and prognosis were acquired by means of medical record consultation and telephone conversation.

### Multiplex ligation-dependent probe amplification (MLPA)

Genomic DNA were extracted from formalin-fixed, paraffin-embedded (FFPE) blocks using QIAamp DNA FFPE Tissue Kit (Qiagen, Valencia, CA). Then DNA copy number quantification and MYD88 L265P mutation were detected using MLPA kit (MRC-Holland, Netherlands). The PCR products were detected on an ABI 3500 Genetic Analyzer (Applied Biosystems, USA), and the final result were analyzed using Coffalyser 9.4 software. The relative peak ratio (PRR) of probe larger than 1.3 was defined as amplification, and less than 0.7 was defined as deletion (see Additional file 2). Genes which had two or more probes covering two different exomes were put into final analysis, including *JAK2*, *PD-L2*, *MDM2, REL, PUS10, BCL2, NFATC1, SPIB, FOXP1, NFKBIZ, BCL6, PRDM1, TNFAIP3, CDKN2A, PTEN, ING1* and *TP53* [6]. The details of MLPA probes of driver genes in DLBCL are shown in the online supporting material (see Additional file 3). True amplification of one gene was regarded only when all probes of this gene exhibited amplification, and vice versa (see Additional file 2).

MYD88 L265P mutation was identified when the probe had a high peak. MYD88 wildtype didn’t show any peak (see Additional file 2).

### Immunohistochemistry (IHC) staining of PD-L1(22C3)

IHC staining was performed on Dako Autostainer Link 48 (ASL48) platform. Each FFPE block were cut at a thickness of 4-μm, and then deparaffinized. Antigen retrieval were performed using the EnVision™ FLEX Target Retrieval Solution at low pH 6.0. Monoclonal PD-L1 (clone 22C3, Dako) were used as primary antibody, followed by incubation with EnVision™ FLEX+ Mouse LINKER, and then EnVision™ FLEX HRP reagent. Finally, the IHC was visualized by EnVision™ FLEX DAB. Each IHC slide contained a positive control (Lung carcinoma).

IHC score of PD-L1 were calculated by multiplying the percentage of positive cells with mean intensity (0, no staining, 1, weak staining, 2, moderate staining; 3, strong staining), which was reported in previous study [5]. The results were evaluated by an experienced hematopathologist (Xuemin).

### Statistical analysis

The differences of clinicopathological characteristics among different groups were analyzed using Chi-square test, Fisher exact test or Kruskal-Wallis rank sum test. PD-L1 IHC score between different groups was analyzed using Wilcoxon test. Overall survival (OS) and progress-free survival (PFS) times were defined from the date of pathologic diagnosis to the date of the event or the last follow-up. The hazard ratio (HR) of each parameter was calculated by univariate Cox proportional regression analysis firstly, in which parameters with *p* < 0.05 were evaluated together using multivariate Cox proportional regression analysis. The survival curve were made according to Kaplan–Meier procedure. The day of last follow-up was March 1st 2019. All statistical analysis were two sided and *p* < 0.05 was defined as significance.

Unsupervised hierarchical clustering was carried out using Euclidean distance and complete method. Heatmap was plot using pheatmap package.

All above statistical analyses were run in R 3.4.1 statistic software.

## Results

### Unsupervised hierarchical clustering of CNAs of driver genes and its survival analysis in DLBCL and PMBCL patients

**Fig. 1:**
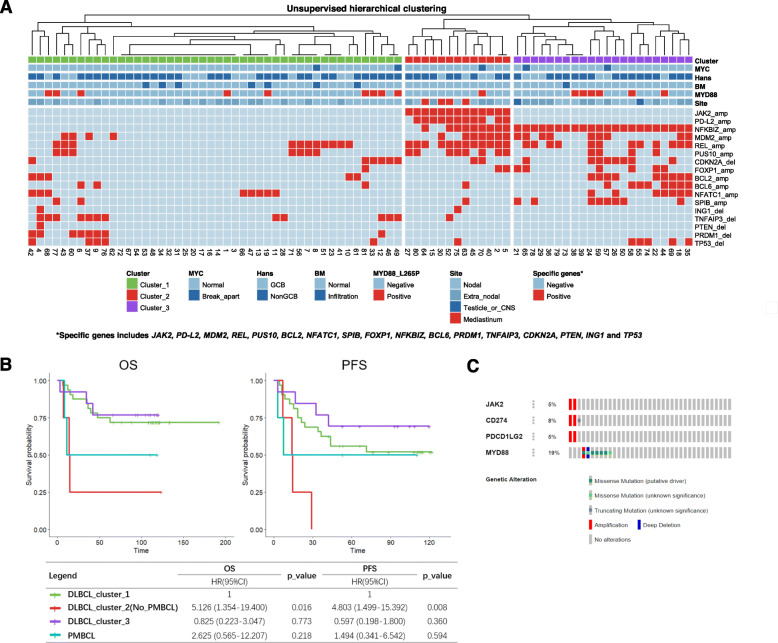
Heatmap and survival analysis based on unsupervised hierarchical clustering, and status of *JAK2*/PD-L1 amplification and MYD88 mutation in TCGA dataset. **a**, Heatmap of CNA-based profiles of driver genes in DLBCL and PMBCL by using unsupervised hierarchical clustering. **b**, Survival curves and cox-regression analysis of OS and PFS among three CNA-based clusters after RCHOP-like treatment. **c**, Status of amplifications of *JAK2*, *PD-L1*(*CD274*) and *PD-L2*(*PDCD1LG2*), and mutation of MYD88 in DLBCL (TCGA, PanCancer Atlas) from cBioPortal [8, 9]

Based on array CGH, Lenz G et al. study previously identified specific CNAs in PMBCL which were different from ABC and GCB of DLBCL [6]. ABC DLBCLs often have CNAs in *FOXP1, NFKBIZ, CDKN2A, CDKN2B, INF4a, BCL2, NFATC1* and *SPIB*, while GCB DLBCLs frequently harbor CNAs in *REL, PTEN, MDM2, MIHG1* and *ING1*. PMBCL often demonstrate CNAs of *JAK2* and *PD-L2* [6]. Using unsupervised hierarchical clustering, we explored the CNA-based pattern of these genes in DLBCL and PMBCL. The result showed that a small group of DLBCL (10.5%, 8/76) was clustered together with PMBCL as Cluster_2, with amplification of *JAK2* (100%,8/8) and *PD-L2* (75.0%%,6/8)(Fig. 1a). This subgroup of DLBCL occurred at the site of cervical lymph node (3 cases), gastrointestinal tract (3 cases), nasal cavity (1 case) and spleen (1 cases) (Fig. 1a, Table 1)(Additional File 4). The frequency of *JAK2* and *PD-L2* amplification in the whole cohort of DLBCL were 10.5% (8/76) and 7.9% (6/76), while both of them were 100% in PMBCL (Fig. 1a) (see Additional file 1). Meanwhile, all cases in Cluster_3 harbored amplification of NFKBIZ which is essential for NF-κB activation in ABC DLBCL [10], but no amplification of NFKBIZ was found in Cluster_1.

**Table 1:** The clinicopathological characteristics of 8 DLBCL with *JAK2/PD-L2* Amplification and 5 PMBCL

No.	Diagnosis	Site	Sex	Age	IPI _risk	Hans algorithm	MYC_ Break-apart	MYD88_ L265P	***JAK2***_ amp	***PD-L2***_ amp
1	DLBCL	Spleen	Female	49	low	Non_GCB	Normal	–	+	+
2	DLBCL	Cervical lymph node	Male	45	low_intermediate	Non_GCB	Normal	–	+	+
3	DLBCL	Stomach	Female	34	low_intermediate	Non_GCB	Normal	–	+	+
4	DLBCL	Stomach	Male	53	low	Non_GCB	Normal	–	+	–
5	DLBCL	Colon	Male	39	high	GCB	Normal	–	+	–
6	DLBCL	Cervical lymph node	Female	27	high_intermediate	Non_GCB	Normal	–	+	+
7	DLBCL	Nasal cavity	Male	63	high_intermediate	GCB	Break_apart	+	+	+
8	DLBCL	Cervical lymph node	Female	70	low_intermediate	Non_GCB	Normal	–	+	+
9	PMBCL	Cervical lymph node	Male	27	low	NA	Normal	–	+	+
10	PMBCL	Mediastinum	Female	19	low	NA	Normal	–	+	+
11	PMBCL	Mediastinum	Female	36	low	NA	Normal	–	+	+
12	PMBCL	Mediastinum	Female	31	high_intermediate	NA	Normal	–	+	+
13	PMBCL	Mediastinum	Male	22	low	NA	Normal	–	+	+

*NA* not applicable

As to survival, DLBCL in Cluster_2 demonstrated significant worse OS (*p* = 0.016) and PFS (*p* = 0.008) as compared with DLBCL in Cluster_1(Fig. 1b). However, Cluster_1 and Cluster_3 didn’t reveal significant difference in survival (Fig. 1b). We also analyzed the OS and PFS between DLBCL with and without JAK2/PD-L2 amplification, and got statistical significance (see Additional file 5).

Of note, we found that DLBCL in Cluster_2 enriched for *JAK2/PD-L2* amplification had less frequency of MYD88_L265P mutation (12.5%, 1/8) (Fig. 1a, Table 4), which was supported by the Cancer Genome Atlas data (TCGA, PanCancer Atlas) from cBioPortal [8, 9] (Fig. 1c).

### *JAK2/PD-L2* amplification identify a distinctive CNA-based pattern of DLBCL similar to that of PMBCL

**Fig. 2:**
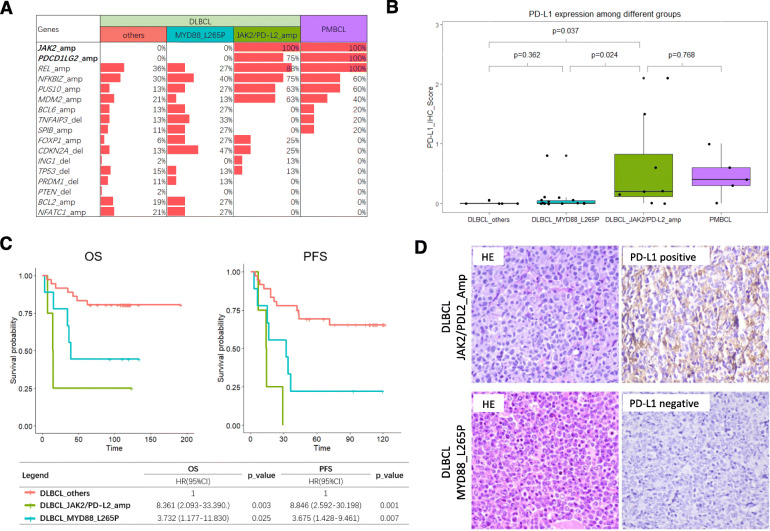
Comparison of CNA-based pattern, PD-L1 expression and survival analysis among PMBCL and three subgroups of DLBCL. **a**, Comparison of CNA-based patterns of driver genes among PMBCL and three subgroups of DLBCL according to the status of *JAK2/PD-L2* amplification and MYD88 L265P mutation. **b**, Comparison of PD-L1 expression (IHC score) among PMBCL and three subgroups of DLBCL. **c**, Survival curves and cox-regression analysis of OS and PFS among three subgroups of DLBCL after RCHOP-like treatment. **d**, Representative images of HE(× 200) and PD-L1(× 200) IHC in DLBCL_JAK2/PD-L2_amp and DLBCL_ MYD88_L265P

Since DLBCL with *JAK2/PD-L2* amplification had less frequency of MYD88 L265P mutation, our study separated DLBCL patients into three subgroups: DLBCL with *JAK2/PD-L2* amplification (DLBCL_JAK2/PD-L2_amp), DLBCL with MYD88 L265P mutation (DLBCL_MYD88_L265P), and DLBCL without *JAK2/PD-L2* amplification nor MYD88_L265P mutation (DLBCL_others) (Fig. 2a). Based on the unsupervised cluster result (Fig. 1a), one patient who had both JAK2/PD-L2 amplification and MYD88 L265P mutation was clustered into Cluster_2. Therefore, this patient was put into DLBCL_JAK2/PD-L2_amp subgroup accordingly. We also analyzed the data when this case was included in DLBCL_MYD88_L265P subgroup, and got the similar result (see Additional file 6).

Unlike DLBCL_MYD88_L265P and DLBCL_others, DLBCL_JAK2/PD-L2_amp showed a distinctive pattern similar to that of PMBCL, with high frequency of REL and NFKBIZ amplifications, but no amplification of *BCL2* and *NFATC1* and no deletion of PRDM1 was found (Fig. 2a).

With respect to clinicopathologic characteristics, DLBCL_JAK2/PD-L2_amp tend to be younger than DLBCL_MYD88_L265P (*p* = 0.003) (Table 2). While, Hans model, international prognostic index (IPI) risk category and MYC break-apart didn’t show any significant differences (Table 2).

**Table 2:** Comparison of characteristics among PMBL and three subgroups of DLBCL

	DLBCL			PMBCL	*p*_value (χ2 test)
	Others	MYD88_L265P	JAK2/PD-L2_amp		
Patients	53 (65.43%)	15 (18.52%)	8 (9.88%)	5 (6.17%)	
Age					0.003*
median (range)	57 (23–80)	57 (31–88)	47 (27–70)	27 (19–36)	
BM					0.520
Negative	46 (86.79%)	14 (93.33%)	8 (100.00%)	5 (100.00%)	
Positive	7 (13.21%)	1 (6.67%)	0 (00.00%)	0 (00.00%)	
IHC Hans’ algorithm					0.806
GCB	16 (30.19%)	3 (20.00%)	2 (25.00%)	2 (40.00%)	
Non-GCB	37 (69.81%)	12 (80.00%)	6 (75.00%)	3 (60.00%)	
IPI					0.522
Low-risk	26 (49.06%)	7 (46.67%)	2 (25.00%)	4 (80.00%)	
Low_intermediate	12 (22.64%)	5 (33.33%)	3 (37.50%)	0 (00.00%)	
High_intermediate	7 (13.21%)	3 (20.00%)	2 (25.00%)	1 (20.00%)	
High	8 (15.09%)	0 (00.00%)	1 (12.50%)	0 (00.00%)	
MYC Breakapart					0.822
Negative	50 (94.34%)	14 (93.33%)	7 (87.50%)	5 (100.00%)	
Positive	3 (05.66%)	1 (06.67%)	1 (12.50%)	0 (00.00%)	

* Kruskal-Wallis rank sum test

### PD-L1 expression in DLBCL with *JAK2/PD-L2* amplification was significantly higher than that in DLBCL with MYD88 L265P mutation

Totally, 32 cases were performed PD-L1 (22C3) IHC detection, including DLBCL_MYD88_L265P (14 cases), DLBCL_JAK2/PD-L2_amp (8 cases), DLBCL_others (5 cases) and PMBCL (5 cases). The result showed that PD-L1 expression in DLBCL_JAK2/PD-L2_amp was significantly higher than that in DLBCL_MYD88_L265P (*p* = 0.024) and DLBCL_others (*p* = 0.037) (Fig. 2b and d). While no significant difference was found between DLBCL_JAK2/PD-L2_amp and PMBCL (*p* = 0.768) (Fig. 2b).

### *JAK2/PD-L2* amplification identify a subgroup of DLBCL with unfavorable survival outcome similar to that of MYD88 L265P mutation

Trying to explore the survival indication of *JAK2/PD-L2* amplification and MYD88 L265P mutation, 49 cases of DLBCLs who received RCHOP-like regiment with or without surgical resection were enrolled to performed cox proportional regression analysis of OS and PFS. The median follow-up time was 108 months (range, 3–192 months).

In the univariate analysis, as compared with DLBCL_others, DLBCLs with MYD88 L265P mutation had significantly worse OS and PFS (*p* = 0.025 and 0.007, respectively), and the same to DLBCLs with *JAK2/PD-L2* amplification (*p* = 0.003 and 0.001, respectively). Meanwhile, IPI risk category were significantly associated with OS and PFS (Fig. 2c, Tables 3 and 4).

**Table 3:** OS in DLBCL treated by RCHOP-like regime

	OS	***p***_value	OS	***p***_value
	HR_U(95%CI)		HR_M(95%CI)	
**Age**				
< 60	1			
≥ 60	3.449 (1.177–10.102)	0.024*		
**BM**				
Negative	1			
Positive	0.918 (0.121–6.985)	0.934		
**Site**				
Extranodal	1			
Nodal	0.736 (0.267–2.029)	0.553		
**IHC Hans’ algorithm**				
GCB	1			
Non-GCB	0.935 (0.320–2.738)	0.903		
**MYC FISH Breakapart**				
Negative	1			
Positive	2.226 (0.291–17.011)	0.440		
**IPI risk category**				
Low	1		1	
Low_intermediate	1.438 (0.263–7.853)	0.675	0.955 (0.171–5.797)	0.996
High_intermediate	6.223 (1.540–25.147)	0.010	4.342 (1.031–18.297)	0.045
High	7.006 (1.875–26.184)	0.004	12.955 (2.946–56.971)	0.001
**Therapy**				
RCHOP	1			
Resection & RCHOP	0.378 (0.085–1.674)	0.200		
**CNA_based_cluster**				
Cluster_1	1			
Cluster_2	5.432 (1.426–20.695)	0.013		
Cluster_3	0.821 (0.222–3.032)	0.767		
**Three subgroups of DLBCL**				
DLBCL_others	1		1	
DLBCL_JAK2/PD-L2_amp	8.361 (2.093–33.394)	0.003	9.558 (1.921–47.560)	0.006
DLBCL_MYD88_L265P	3.732 (1.177–11.830)	0.025	7.706 (1.838–32.314)	0.005

*HR_U* hazard ratio by univariate analysis, *HR_M* hazard ratio by multivariate analysis

* Because age was contained in the IPI, thus it wasn’t put into multivariate analysis

**Table 4:** PFS in DLBCL treated by RCHOP-like regime

	PFS	***P***_value	PFS	***P***_value
	HR_U(95%CI)		HR_M(95%CI)	
**Age**				
< 60	1			
≥ 60	3.210 (1.376–7.489)	0.007*		
**BM**				
Negative	1			
Positive	2.764 (0.811–9.425)	0.104		
**Site**				
Extranodal	1			
Nodal	1.104 (0.484–2.520)	0.813		
**IHC Hans’ algorithm**				
GCB	1			
Non-GCB	0.731 (0.316–1.690)	0.464		
**MYC Breakapart**				
Negative	1			
Positive	1.235 (0.166–9.187)	0.836		
**IPI**				
Low	1		1	
Low_intermediate	1.713 (0.514–5.703)	0.380	1.836 (0.540–6.248)	0.331
High_intermediate	3.103 (0.928–10.375)	0.066	2.291 (0.658–7.982)	0.193
High	9.329 (3.239–26.869)	< 0.001	15.382 (4.722–50.104)	< 0.001
**Therapy**				
RCHOP	1			
Resection & RCHOP	0.611 (0.226–1.648)	0.330		
**CNA_based_clusters**				
Cluster_1	1			
Cluster_2	5.344 (1.639–17.427)	0.005		
Cluster_3	0.593 (0.197–1.787)	0.353		
**Three subgroups of DLBCL**				
DLBCL_others	1		1	
DLBCL_ JAK2/PD-L2_amp	8.846 (2.592–30.198)	0.001	9.246 (2.390–35.774)	0.001
DLBCL_ MYD88_L265P	3.675 (1.428–9.461)	0.007	6.150 (2.153–17.569)	0.001

*HR_U* hazard ratio by univariate analysis, *HR_M* hazard ratio by multivariate analysis

* Because age was contained in the IPI, thus it wasn’t put into multivariate analysis

In the multivariate analysis, IPI risk category and three subgroups of DLBCL were put into analysis. As compared with DLBCL_others, DLBCL with MYD88 L265P mutation still showed poor OS and PFS (*p* = 0.005 and 0.001, respectively), and the same to DLBCL with *JAK2/PD-L2* amplification for PFS and OS (*p* = 0.006 and 0.001, respectively). Meanwhile, IPI risk category was still an independent risk predictors for OS and PFS (Fig. 2c, Tables 3 and 4).

### Either *JAK2/PD-L2* amplification or MYD88 L265P mutation are frequently seen in relapse/refractory DLBCL with PFS less than 2 years

DLBCL with PFS less than 2 years was defined as primary relapse/refractory cases. Among these cases who treated by RCHOP-like regime, the frequency of *JAK2* and *PD-L2* amplification were 20%(3/15) and 13.3% (2/15). Meanwhile, the frequency of MYD88 L265P mutation were 33.3% (5/15). DLBCL with either *JAK2/PD-L2* amplification or MYD88 L265P accounted for 46.7% (7/15).

## Discussion

DLBCL presents with a wide spectrum of genetic aberration. Recently, Shi et al. study exhibited *PD-L2* amplification in 75% PMBCL and 0% of DLBCL [11]. Chapuy et al. demonstrated 15% of 9p24.1 amplification in DLBCL [2]. Meanwhile, DLBCL with PD-L1 gene alterations was identified as a unique biological subgroup, having high risk features [3]. Y Wang et al. study demonstrated that 10% of DLBCL had CNA of 9p24.1, with gene expression and mutation profiles that were similar to those of PMBCL [5]. In our study, by using unsupervised hierarchical clustering, 10.5% (8/76) cases of DLBCL were clustered together with PMBCL as Cluster_2, indicating that they shared recurrent CNAs. They were enriched for *JAK2* amplification and *PD-L2* amplification (Fig. 1a).

Using Hans model, most of DLBCL in Cluster_2 were non-GCB (75%, 6/8), and tend to be younger than other groups of DLBCL (Table 2), which was consistent with prior study [5]. Therefore, coupled with Y Wang et al. study, we confirmed that DLBCL with *JAK2/PD-L2* amplification is a unique subgroup resembling the PMBCL with respect to CNA pattern.

With regard to survival, increasingly data exhibited that the suppression of immune surveillance in DLBCL was associated with poor survival. Godfrey J et al. study has demonstrated that DLBCL with PD-L1 gene alterations showed high risk features [3]. Meta-analysis also showed that PD-L1 expression was associated with poor OS and adverse clinicopathologic features in DLBCL [12].

In Y Wang et al. study, 10% of DLBCL harbored CNA of 9p24.1, of which 65% were gains and 35% were amplifications [5]. And, as compared with those who have no gain of 9p24.1, DLBCL with 9p24 amplification had a trend of better EFS, while patients with only gain tend to have worse prognosis [5]. Unfortunately, they didn’t show any statistical significance [5]. In our study, 10.5% (8/76) of DLBCL were found that had CNA of JAK2. When JAK2 CNA was separated into gain (MLPA value between 1.7–2.0) and amplification (MLPA value > 2.0) as described [13], 50%(4/8) cases in DLBCL_JAK2/PD-L2_amp group were found that had JAK2 gain, which was slightly lower than that in Wang J et al. study [5] (as shown in Additional File 7). And both DLBCL with JAK2 gain and with amplification demonstrated significant poor prognosis as compared with rest of DLBCL (as shown in Additional File 7). More interesting, unlike Y Wang et al. study [5], 5 cases of PMBCL were included in our study as control, all of which demonstrating JAK2 gains, rather than amplifications (as shown in Additional File 7).

Of note, we found that DLBCL in Cluster_2 enriched for *JAK2/PD-L2* amplification had less frequency of MYD88_L265P mutation (12.5%, 1/8) (Fig. 1a, Table 4), which was supported by the Cancer Genome Atlas data (TCGA, PanCancer Atlas) from cBioPortal [8, 9] (Fig. 1c).

MYD88 L265P is a poor indicator of survival for DLBCL [14] which may lead to primary refractory/relapsed disease. This is a gain-of-function driver mutation, occurring in 10% ~ 24% of DLBCL, but absent in PMBCL [14–16]. In our study, the frequency of MYD88 L265P in DLBCL and PMBCL were 21.1%(16/76) and 0%(0/5), which were in line with prior studies [14–16]. Of great interest, MYD88 L265P mutation occurred less frequently in Cluster_2 (12.5%, 1/8), which was supported by the data (TCGA, PanCancer Atlas) from cBioPortal [8, 9]. Thus, when we divided DLBCL patients into three subgroups (DLBCL_JAK2/PD-L2_amp, DLBCL_MYD88_L265P and DLBCL_others, both DLBCL_JAK2/PD-L2_amp and DLBCL_MYD88_L265P demonstrated dismal OS and PFS with a median follow-up of 9 "years, as compared with DLBCL_others. Therefore, DLBCL with *JAK2/PD-L2* amplification was identified as a poor survival subgroup that is similar to DLBCL with MYD88 L265P mutation.

Meanwhile, we also compared the CNA patterns of driver genes among DLBCL_JAK2/PD-L2_amp, DLBCL_MYD88_L265P, DLBCL_others and PMBCL. DLBCL_JAK2/PD-L2_amp showed a distinctive pattern similar to PMBCL, with high frequency of *REL* and *NFKBIZ* amplifications, but no amplification of *BCL2* and *NFATC1* and no deletion of *PRDM1* was found. The profile of DLBCL_MYD88_L265P was closed to DLBCL_others, showing relatively high frequency of *CDKN2A* deletion, *NFATC1* amplification and *BCL2* amplification.

In our study, 75.0%(6/8) of DLBCL_JAK2/PD-L2_amp harbored both *JAK2* and *PD-L2* amplifications simultaneously, indicating that they may also have the PD-L1 amplification, because *PD-L1* located in the middle of *JAK2* and *PD-L2* at 9p24.1. Thus, we hypothesized that PD-L1 expression would be upregulated in this subgroup. As what we expected, using PD-L1 (22C3) IHC detection, PD-L1 expression in DLBCL_JAK2/PD-L2_amp was significantly higher than that in DLBCL_MYD88_L265P (*p* = 0.024) and DLBCL_others (*p* = 0.037) (Fig. 2b and d), but not in PMBCL (*p* = 0.768) (Fig. 2b). Meanwhile, PD-L1 expression could be enhanced not only by *PD-L1* amplification but also by *JAK2* activation [4, 17]. Therefore, DLBCL with *JAK2/PD-L2* amplification was confirmed as an unique subtype that is different from DLBCL with MYD88 L265P and others.

Objective response rates (ORR) of PD-1 blockade therapy was 10–36% in unselected patients with relapsed/refractory DLBCL [18, 19]. The wide spectrum of ORR may be due to high heterogeneity of this subgroup. Ansell SM et al. study demonstrated 19% patients with 9p24.1 alteration in relapsed/refractory DLBCL [18]. In our cohort, the frequency of *JAK2* and *PD-L2* amplification in relapsed/refractory DLBCL were 20% and 13.3%, which were within the range of ORR in the prior studies [18, 19]. While, 33.3% (5/15) patients were found that had MYD88 L265P mutation who may not be suitable for anti-PD-1 therapy. Thus, the genetic analysis in refractory/relapsed DLBCL is required for future therapy selection to increase the ORR of immune checkpoint inhibitors.

*JAK2* amplification could augment the expression of itself and PD-1 ligands (PD-L1 and *PD-L2*), enhancing the sensitivity to JAK2 kinase inhibitor [4]. Chemical JAK2 inhibition could reduce the RNA transcription and protein expression of PD-L1 [20]. Thus, selective inhibition of JAK2 would be a valuable complementary therapy for PD-L1 blockade.

## Conclusions

DLBCL with amplification of *JAK2/PD-L2* exhibits PMBCL-like CNAs pattern, and demonstrates unfavorable outcome resembling those with MYD88 L265P mutation. Thus, it is essential to identify this subgroup of DLBCL who may acquire more benefits from the JAK2 and PD-L1 signaling inhibition, and *JAK2* amplification detection by MLPA would be feasible in routine practice. Meanwhile, the difference of survival outcome between our study and Wang J et al. study indicated that PMBCL-like DLBCL suggested by 9p24.1 CNA could be an intermixed subgroup, which required further exploration.

## Supplementary information

**Additional file 1.** MLPA results and clinical follow-up data. The clinicopathological characteristics, clinical follow-up data and MLPA results are showed in this file.

**Additional file 2: Figure S1.** Representative results of MLPA. Representative results of MLPA are showed in this figure.

**Additional file 3: Table S1.** The details of MLPA probes of genes in DLBCL. The locations and lengths of MLPA probes of genes are showed in this table.

**Additional file 4.** The detailed information of DLBCL with JAK2/PD-L2 amplification. The detailed data about clinicopathological characteristics, morphology, immunohistochemistry and treatments of DLBCL with JAK2/PD-L2 amplification are showed in this file.

**Additional file 5: Figure S2.** the OS and PFS of DLBCL with or without JAK2/PD-L2_amp. the OS and PFS of DLBCL with or without JAK2/PD-L2_amp.

**Additional file 6: Figure S3.** Comparison of CNA-based pattern and their survival outcome among PMBCL and three subgroups of DLBCL (one case of DLBCL with JAK2/PD-L2 amplification and MYD88 L265P mutation were included in DLBCL_MYD88_L265P group). **a,** Comparison of CNA-based patterns of driver genes among PMBCL and three subgroups of DLBCL according to the status of *JAK2/PD-L2* amplification and MYD88 L265P mutation. **b,** Survival curves and cox-regression analysis of OS and PFS among three subgroups of DLBCL after RCHOP-like treatment.

**Additional file 7: Figure S4.** The frequencies of JAK2 gain and amplification, and their survival analysis. **a.** The frequencies of JAK2 gain and amplification in DLBCL_JAK2/PD-L2_amp and PMBCL. **b.** the OS and PFS of DLBCL with JAK2 gain or with JAK2 amplification.

## Data Availability

All data generated or analyzed during this study are included in this published article and its supplementary information files.

## Abbreviations

DLBCL: Diffuse large B-cell lymphoma
PMBCL: Primary mediastinal large B-cell lymphoma
MLPA: Multiplex ligation-dependent probe amplification
TCGA: The Cancer Genome Atlas
IPI: International Prognostic Index
FFPE: Formalin-fixed, paraffin-embedded
OS: Overall survival
PFS: Progress-free survival
HR: Hazard ratio
