# Supplementary material for: DLBCL with amplification of JAK2/PD-L2 exhibits PMBCL-like CNA pattern and worse clinical outcome resembling those with MYD88 L265P mutation
Source: BMC Cancer. 2020 Aug 27;20:816. doi: 10.1186/s12885-020-07293-3 (PMC7450805; doi:10.1186/s12885-020-07293-3)
Supplement: Supplementary file 2 — Additional file 2: Figure S1. Representative results of MLPA. Representative results of MLPA are showed in this figure. [file 12885_2020_7293_MOESM2_ESM.ppt]

## Slide 1
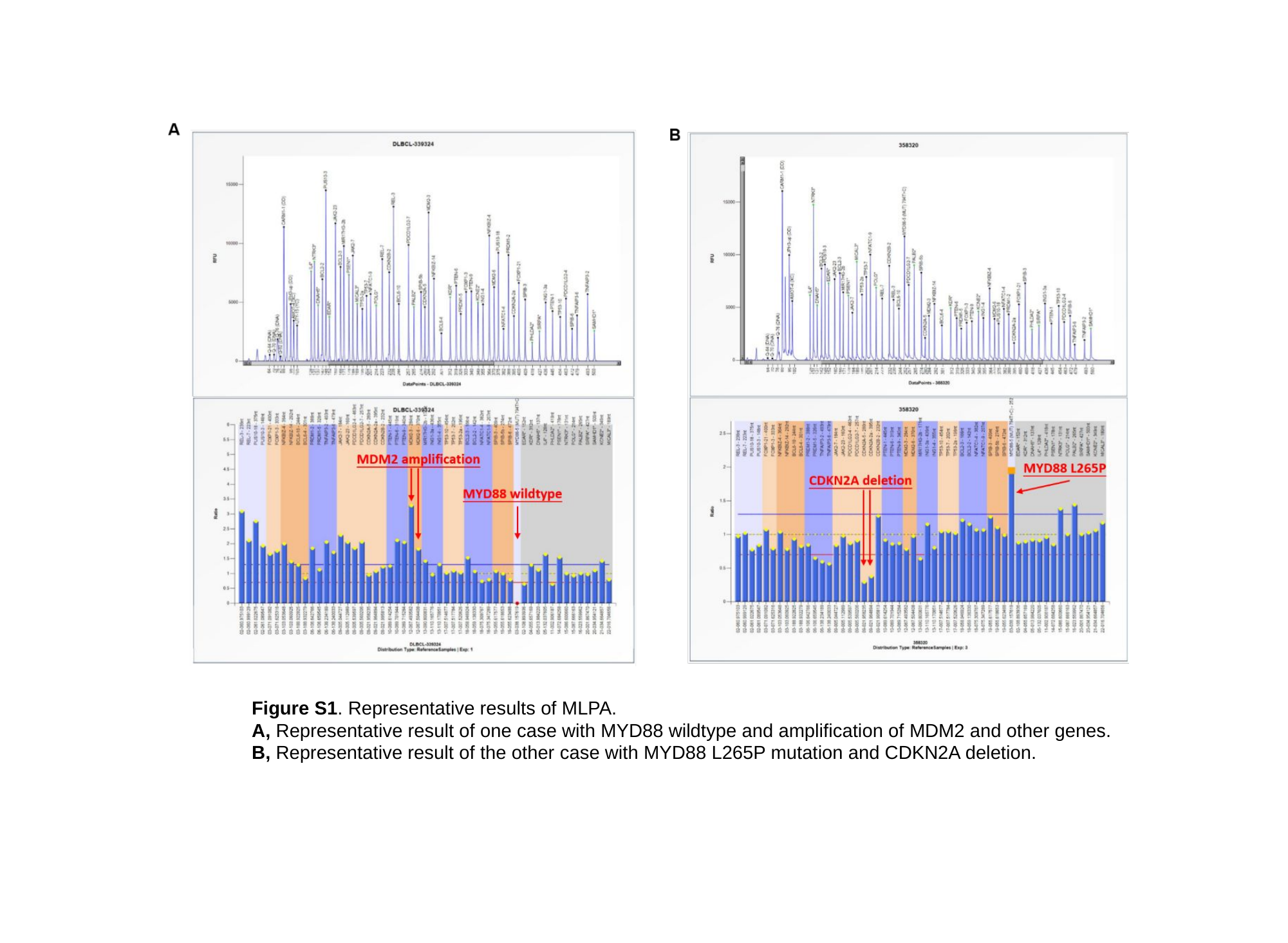

Figure S1. Representative results of MLPA.
A, Representative result of one case with MYD88 wildtype and amplification of MDM2 and other genes.
B, Representative result of the other case with MYD88 L265P mutation and CDKN2A deletion.
