# Supplementary material for: DLBCL with amplification of JAK2/PD-L2 exhibits PMBCL-like CNA pattern and worse clinical outcome resembling those with MYD88 L265P mutation
Source: BMC Cancer. 2020 Aug 27;20:816. doi: 10.1186/s12885-020-07293-3 (PMC7450805; doi:10.1186/s12885-020-07293-3)
Supplement: Supplementary file 3 — Additional file 3: Table S1. The details of MLPA probes of genes in DLBCL. The locations and lengths of MLPA probes of genes are showed in this table. [file 12885_2020_7293_MOESM3_ESM.doc]

**Table S1. The details of MLPA probes of genes in DLBCL**

| **D [nt]** | **Gene** | **Exon** | **Chr.band** | **hg18 loc.** |
| --- | --- | --- | --- | --- |
| **238** | REL | 3 | 02p16.1 | 02-060.975103 |
| **223** | REL | 7 | 02p16.1 | 02-060.999129 |
| **375** | PUS10 | 18 | 02p16.1 | 02-061.022675 |
| **148** | PUS10 | 3 | 02p16.1 | 02-061.089547 |
| **400** | FOXP1 | 21 | 03p13 | 03-071.091082 |
| **333** | FOXP1 | 3 | 03p13 | 03-071.625316 |
| **364** | NFKBIZ | 4 | 03q12.3 | 03-103.053648 |
| **292** | NFKBIZ | 14 | 03q12.3 | 03-103.060925 |
| **244** | BCL6 | 10 | 03q27.3 | 03-188.922825 |
| **301** | BCL6 | 4 | 03q27.3 | 03-188.932279 |
| **388** | PRDM1 | 2 | 06q21 | 06-106.642766 |
| **326** | PRDM1 | 5 | 06q21 | 06-106.659545 |
| **493** | TNFAIP3 | 2 | 06q23.3 | 06-138.234189 |
| **479** | TNFAIP3 | 6 | 06q23.3 | 06-138.240033 |
| **184** | JAK2 | 7 | 09p24.1 | 09-005.044727 |
| **160** | JAK2 | 23 | 09p24.1 | 09-005.112989 |
| **463** | PDCD1LG2 | 4 | 09p24.1 | 09-005.539597 |
| **257** | PDCD1LG2 | 7 | 09p24.1 | 09-005.560206 |
| **280** | CDKN2A | 5 | 09p21.3 | 09-021.958235 |
| **395** | CDKN2A | 2a | 09p21.3 | 09-021.964894 |
| **445** | PTEN | 1 | 10q23.31 | 10-089.614254 |
| **319** | PTEN | 6 | 10q23.31 | 10-089.701944 |
| **340** | PTEN | 9 | 10q23.31 | 10-089.715284 |
| **284** | MDM2 | 3 | 12q15 | 12-067.493582 |
| **370** | MDM2 | 6 | 12q15 | 12-067.504408 |
| **436** | ING1 | 3a | 13q34 | 13-110.165776 |
| **355** | ING1 | 4 | 13q34 | 13-110.170851 |
| **454** | TP53 | 10 | 17p13.1 | 17-007.514677 |
| **202** | TP53 | 7 | 17p13.1 | 17-007.517784 |
| **196** | TP53 | 2a | 17p13.1 | 17-007.520626 |
| **166** | BCL2 | 3 | 18q21.33 | 18-058.946924 |
| **142** | BCL2 | 2 | 18q21.33 | 18-059.13633 |
| **382** | NFATC1 | 4 | 18q23 | 18-075.309767 |
| **207** | NFATC1 | 9 | 18q23 | 18-075.347289 |
| **409** | SPIB | 3 | 19q13.33 | 19-055.617577 |
| **274** | SPIB | 5b | 19q13.33 | 19-055.618653 |
| **472** | SPIB | 6 | 19q13.33 | 19-055.623488 |
| **252** | MYD88_L265P | 5 | 03p22.2 | 03-038.157619 |
