# Supplementary figures and images for: DLBCL with amplification of JAK2/PD-L2 exhibits PMBCL-like CNA pattern and worse clinical outcome resembling those with MYD88 L265P mutation
Source: BMC Cancer. 2020 Aug 27;20:816. doi: 10.1186/s12885-020-07293-3 (PMC7450805; doi:10.1186/s12885-020-07293-3)

## Slide 1
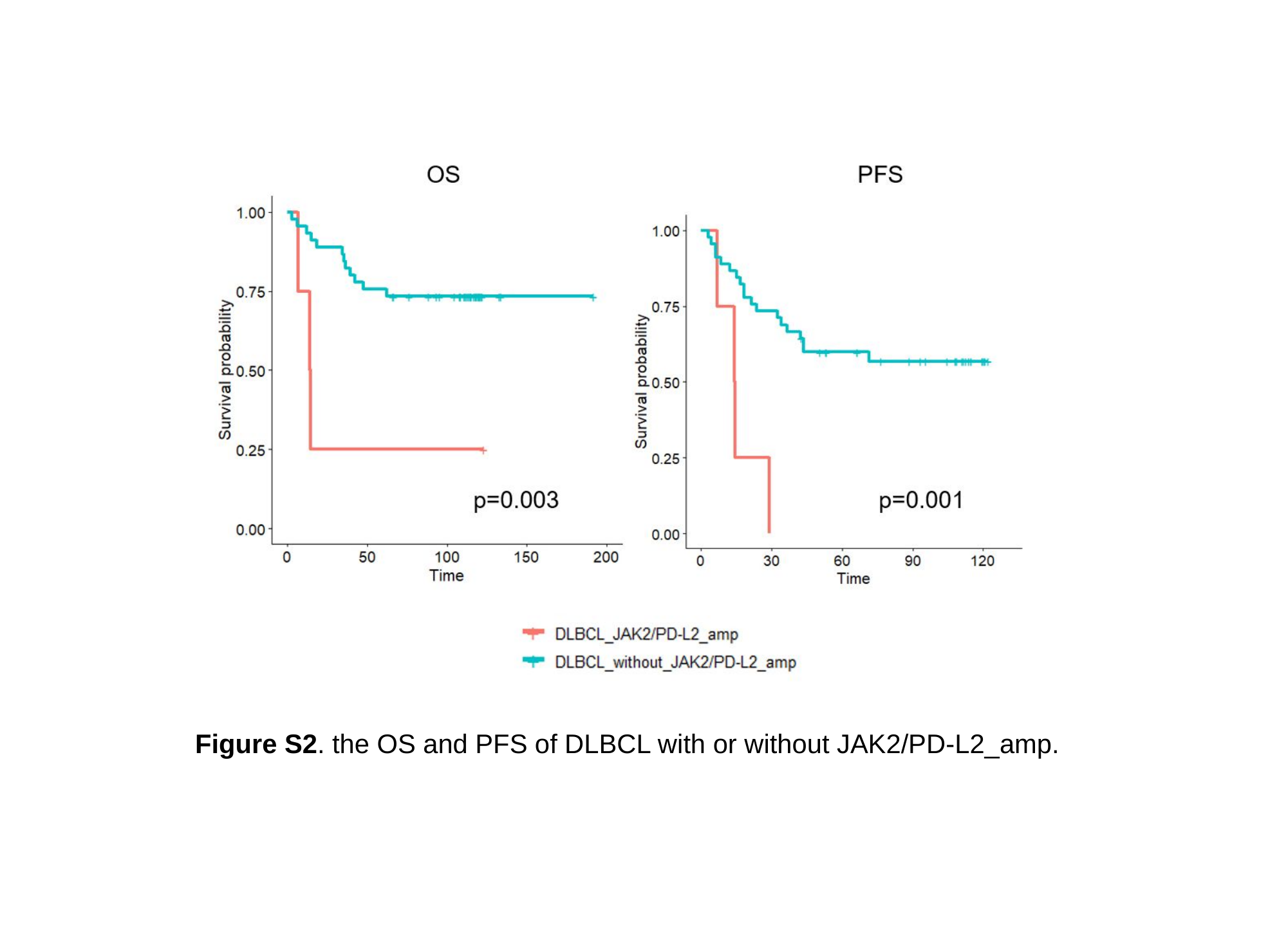

Figure S2. the OS and PFS of DLBCL with or without JAK2/PD-L2_amp.

Supplement: Supplementary file 5 — Additional file 5: Figure S2. the OS and PFS of DLBCL with or without JAK2/PD-L2_amp. the OS and PFS of DLBCL with or without JAK2/PD-L2_amp. [file 12885_2020_7293_MOESM5_ESM.ppt]
