# Supplementary material for: DLBCL with amplification of JAK2/PD-L2 exhibits PMBCL-like CNA pattern and worse clinical outcome resembling those with MYD88 L265P mutation
Source: BMC Cancer. 2020 Aug 27;20:816. doi: 10.1186/s12885-020-07293-3 (PMC7450805; doi:10.1186/s12885-020-07293-3)
Supplement: Supplementary file 6 — Additional file 6: Figure S3. Comparison of CNA-based pattern and their survival outcome among PMBCL and three subgroups of DLBCL (one case of DLBCL with JAK2/PD-L2 amplification and MYD88 L265P mutation were included in DLBCL_MYD88_L265P group). a, Comparison of CNA-based patterns of driver genes among PMBCL and three subgroups of DLBCL according to the status of JAK2/PD-L2 amplification and MYD88 L265P mutation. b, Survival curves and cox-regression analysis of OS and PFS among three subgroups of DLBCL after RCHOP-like treatment. [file 12885_2020_7293_MOESM6_ESM.ppt]

## Slide 1
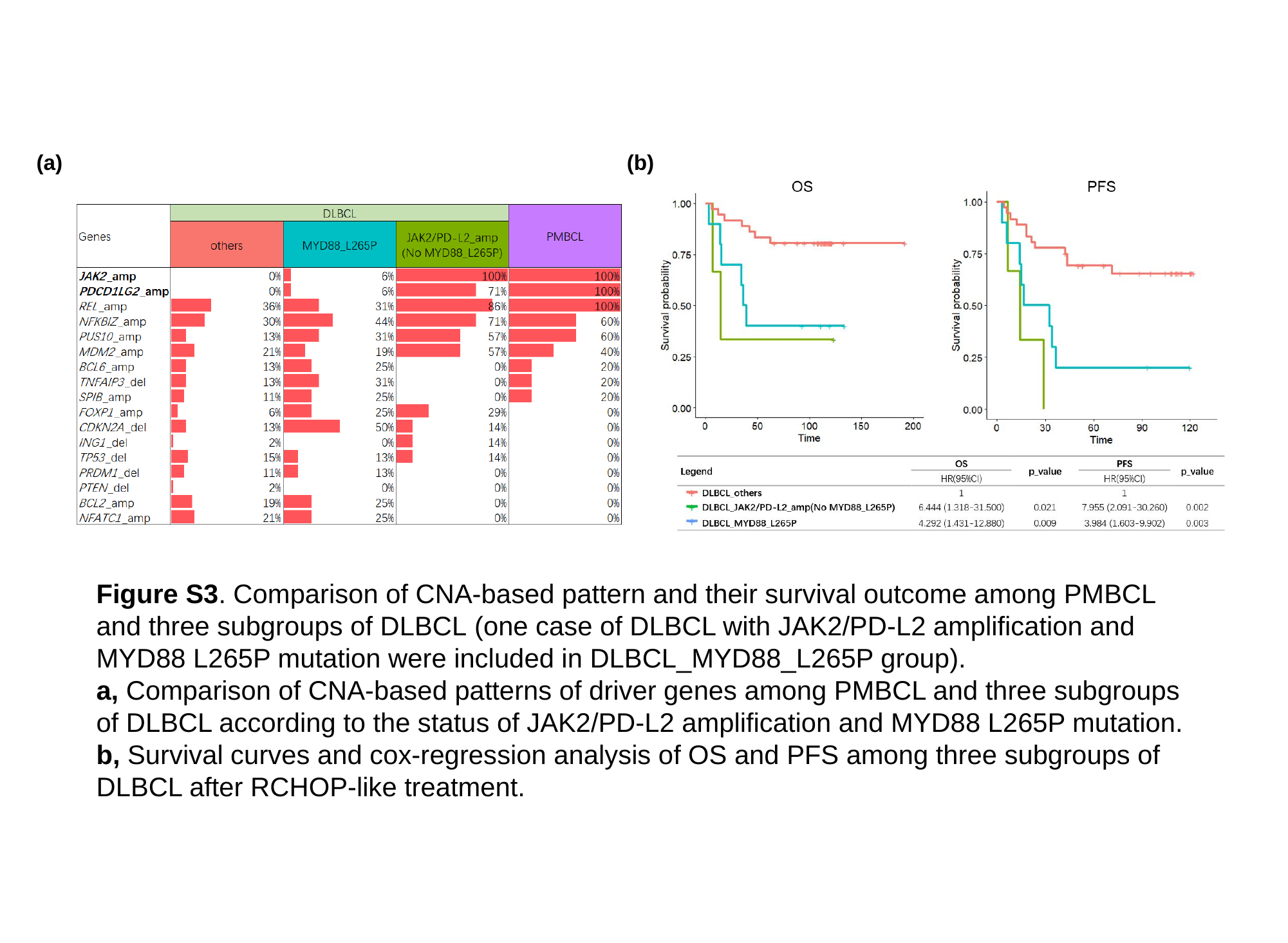

(a)
(b)
Figure S3. Comparison of CNA-based pattern and their survival outcome among PMBCL
and three subgroups of DLBCL (one case of DLBCL with JAK2/PD-L2 amplification and
MYD88 L265P mutation were included in DLBCL_MYD88_L265P group).
a, Comparison of CNA-based patterns of driver genes among PMBCL and three subgroups
of DLBCL according to the status of JAK2/PD-L2 amplification and MYD88 L265P mutation.
b, Survival curves and cox-regression analysis of OS and PFS among three subgroups of
DLBCL after RCHOP-like treatment.
