# Supplementary material for: DLBCL with amplification of JAK2/PD-L2 exhibits PMBCL-like CNA pattern and worse clinical outcome resembling those with MYD88 L265P mutation
Source: BMC Cancer. 2020 Aug 27;20:816. doi: 10.1186/s12885-020-07293-3 (PMC7450805; doi:10.1186/s12885-020-07293-3)
Supplement: Supplementary file 7 — Additional file 7: Figure S4. The frequencies of JAK2 gain and amplification, and their survival analysis. a. The frequencies of JAK2 gain and amplification in DLBCL_JAK2/PD-L2_amp and PMBCL. b. the OS and PFS of DLBCL with JAK2 gain or with JAK2 amplification. [file 12885_2020_7293_MOESM7_ESM.ppt]

## Slide 1
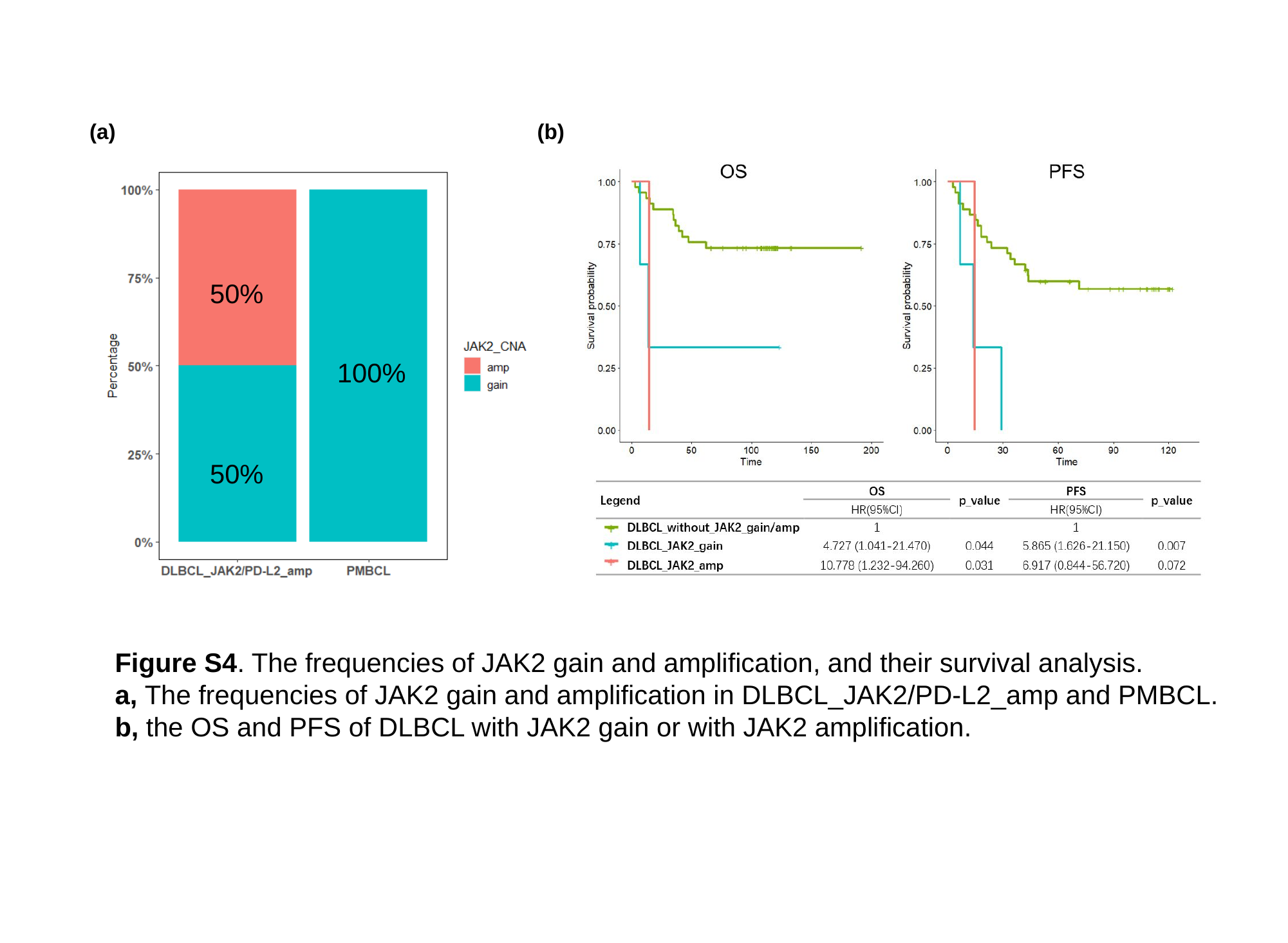

(a)
(b)
50%
100%
50%
Figure S4. The frequencies of JAK2 gain and amplification, and their survival analysis.
a, The frequencies of JAK2 gain and amplification in DLBCL_JAK2/PD-L2_amp and PMBCL.
b, the OS and PFS of DLBCL with JAK2 gain or with JAK2 amplification.
